# Effect of Low Hydroxyapatite Loading Fraction on the Mechanical and Tribological Characteristics of Poly(Methyl Methacrylate) Nanocomposites for Dentures

**DOI:** 10.3390/polym13060857

**Published:** 2021-03-11

**Authors:** Ahmed Fouly, Ahmed Mohamed Mahmoud Ibrahim, El-Sayed M. Sherif, Ahmed M.R. FathEl-Bab, A.H. Badran

**Affiliations:** 1 Research Chair for Tribology, Surface, and Interface Sciences (TSIS), Department of Physics and Astronomy, College of Science, King Saud University, P.O. Box 2455, Riyadh 11451, Saudi Arabia; esherif@ksu.edu.sa; 2 Mechanical Engineering Department, College of Engineering, King Saud University, Riyadh 11421, Saudi Arabia; 3 Department of Production Engineering and Mechanical Design, Faculty of Engineering, Minia University, Minia 61519, Egypt; ahmedkhalifa@mu.edu.eg (A.M.M.I.); ahmed.badran@mu.edu.eg (A.H.B.); 4 Center of Excellence for Research in Engineering Materials (CEREM), King Saud University, P.O. Box 800, Al-Riyadh 11421, Saudi Arabia; 5 Mechatronics and Robotics Engineering Department, School of Innovative Design Engineering, E-JUST, Alexandria 21934, Egypt; ahmed.rashad@ejust.edu.eg

**Keywords:** PMMA nanocomposite, hydroxyapatite nanoparticles, denture base material, low loading fraction

## Abstract

Denture base materials need appropriate mechanical and tribological characteristics to endure different stresses inside the mouth. This study investigates the properties of poly(methyl methacrylate) (PMMA) reinforced with different low loading fractions (0, 0.2, 0.4, 0.6, and 0.8 wt.%) of hydroxyapatite (HA) nanoparticles. HA nanoparticles with different loading fractions are homogenously dispersed in the PMMA matrix through mechanical mixing. The resulting density, Compressive Young’s modulus, compressive yield strength, ductility, fracture toughness, and hardness were evaluated experimentally; the friction coefficient and wear were estimated by rubbing the PMMA/HA nanocomposites against stainless steel and PMMA counterparts. A finite element model was built to determine the wear layer thickness and the stress distribution along the nanocomposite surfaces during the friction process. In addition, the wear mechanisms were elucidated via scanning electron microscopy. The results indicate that increasing the concentration of HA nanoparticles increases the stiffness, compressive yield strength, toughness, ductility, and hardness of the PMMA nanocomposite. Moreover, tribological tests show that increasing the nanoparticle weight fraction considerably decreases the friction coefficient and wear loss.

## 1. Introduction

Suitable mechanical and tribological characteristics allow dentures to endure forces and friction in the mouth [1]. Furthermore, their base materials must be biocompatible and must not undergo chemical reactions potentially harmful to humans. Since 1937, poly(methyl methacrylate) (PMMA) is among the most used materials in denture fabrication due to its light weight, esthetic features, ease of processing and polishing, clinical manipulation, low cost, and stability in the mouth environment [2]. All these advantages have encouraged dentists to utilize it for decades as a denture base and dental restorative polymer [3]. Dentures are traditionally prepared by mixing a prepolymerized PMMA powder with methyl methacrylate (MMA) monomers in the liquid form and pouring the resulting mixture into dental molds [4].

However, PMMA is not always a preferable choice. PMMA has poor surface properties, which prevents its use in frictional applications, and also insufficient mechanical properties (e.g., flexural and impact strengths) [5]. When utilized as a denture base material, PMMA undergoes different stresses, such as shear, tensile, and compressive forces; this can lead to fractures, deep scratches, and increased wear rate, potentially changing the denture base contour and harming the patient [6]. In particular, sudden fractures are a prevalent problem due to the low toughness and brittleness of pure PMMA, disturbing the patients as well as increasing the treatment cost and duration. Darbar et al. [7] documented the types of denture base (made of PMMA) fractures, and they found that 29% of the fractures were in the complete upper dentures and 38% of the remaining fractures were located in the PMMA connectors of upper partial dentures. 

The main requirements for denture base materials are presently appropriate strength, toughness, and wear resistance [8]. To overcome the shortage in the PMMA properties, researchers are trying to enhance its properties through various modifications. For example, the resin has been chemically modified via plasma technology to increase its impact resistance and mixed with agents enhancing its cross-linking [9]. Others have tried to reinforce PMMA with different materials since reinforcing fillers such as particles and fibers can generally improve the properties of a composite [10]. Kanie et al. [11] reported this filler addition as one of the most significant approaches to boost the mechanical characteristics of composites. Asar et al. [12] investigated the effect of adding different metal oxides (TiO_2_, ZrO_2_, and Al_2_O_3_) on the mechanical and physical properties of a heat-cured PMMA resin, demonstrating the enhancement of properties such as fracture toughness; however, some physical effects were clinically undesirable due to its mixture with oral fluids. The effect of utilizing the aggregation of ZrO_2_/Al_2_O_3_ and ZrO_2_/TiO_2_ was also investigated [13], showing the same results as Asar et al. [10]. Based on the same approach, Alhareb et al. [14] studied the fracture toughness, impact strength, and hardness of PMMA when combined with various concentrations of different fillers, that is, nitrile butadiene rubber (NBR) and ceramic fillers such as yttria-stabilized zirconia (YSZ) and Al_2_O_3_ in the presence of a silane agent; all the mechanical properties were improved, and the optimal mixture was 7.5% NBR, 2.5% Al_2_O_3_, and 2.5% YSZ. However, the pure PMMA and its as-obtained composite largely differed in weight due to the high loading of the fillers, especially the ceramic ones.

Nanotechnology has recently invaded the material science field, leading to an evolution in the material properties; thus, scientists have tried to integrate it into dentistry as well [15,16,17]. The nanocomposite characteristics depend on the size, nature, morphology, dispersion, and loading ratio of the nanofiller [18,19,20]. Shakeri et al. [21] investigated the influence of double-modified (organically) nanoclays on the characteristics of the PMMA denture base; adding 0.5 wt.% of this filler improved the flexural modulus, flexural strength, and fracture toughness by 65.8%, 30%, and 32%, respectively. Salahuddin [22] studied the effects of ZnO quantity and morphology on the thermal and mechanical characteristics of PMMA/ZnO nanocomposites, observing a significant improvement in the impact strength when using ZnO nanotubes, while the addition of ZnO nanospheres enhanced the flexural strength.

The tribological and mechanical properties of PMMA reinforced using nanotitania and calcium aluminate with different volume fractions, 1 and 5 vol.%, respectively, have been investigated both theoretically and experimentally [23]. Nabhan et al. [24] studied the effect of adding hybrid nanomaterials (graphene, SiO_2_, and TiO_2_) on the tribological properties of PMMA nanocomposites experimentally. They reported a remarkably enhanced wear resistance when increasing the nanodioxide weight fraction and, in general, observed a gradual increase in the compressive strength, hardness, toughness, and Young’s modulus with the increase in filler volume fraction. Moreover, a noticeable enhancement in the tribological properties (i.e., friction coefficient and wear resistance) was recorded. Yang et al. [25] reinforced PMMA with different contents (0.5–2.5 wt.%) of carbon nanotubes (CNTs). This significantly decreased its friction coefficient and enhanced its wear resistance. However, the application of CNTs is mainly limited by esthetic requirements. Farhan et al. [26] incorporated various fractions (2–5 vol.%) of TiO_2_/ZnO nanoparticles (NPs) into PMMA; the friction coefficient and wear rate decreased with increasing the NP loading fraction and sliding time. Ameer et al. [27] reinforced hot- and cold-cured PMMA with multiwalled carbon nanotubes (MWCNTs). The hardness and wear resistance of the hot-cured resin increased along with the MWCNT loading up to 0.3 wt.%. Moreover, the friction coefficient considerably decreased for both the hot- and cold-cured PMMA.

Hydroxyapatite (HA) is a bioceramic material that has attracted much attention due to its remarkable potential for biomedical applications [28,29]. HA has been widely utilized as a substituting material for damaged bones and teeth [30]. Its compatibility with bone tissue has been experimentally proved, along with its ability to enhance the surface hardness and toughness of composites [31]. HA can enhance the properties of PMMA as a denture base material since it does not undergo allergic nor toxic reactions in the mouth environment [32]. Furthermore, it significantly influences the mechanical properties of polymers when used as a reinforcing material [33]. Bakar et al. [34] studied the effect of reinforcing polyetheretherketone (PEEK) with different fractions (5–40 vol.%) of HA particles on the biological and mechanical behavior of the resulting composites. They reported a considerable dependence of the tensile properties and fatigue life on the HA loading fraction. Converse et al. [35] investigated the mechanical properties of PEEK reinforced with 0–50 vol.% HA whiskers fabricated via compression molding for uniform mixing. The ultimate tensile strength and elastic modulus of the composites could mimic those of the human cortical bone. Zebarjad et al. [36] reinforced PMMA with high fractions of nano-HA (2.5–10 wt.%), decreasing its ultimate and yield compressive strength but enhancing its wear resistance. Chow et al. [37] used high loading fractions (5–20 wt.%) of HA. They reported that the PMMA/HA composite strength deteriorated with the increase in the HA content. Virginia et al. [38] reinforced PMMA with 3–12 wt.% HA. They stated that the wear resistance lessened at high HA contents. All these studies attributed the deterioration in the properties of PMMA/HA composites to the agglomeration of HA particles inside the PMMA matrix. Therefore, many researchers encouraged using a low-loading fraction of the nano-fillers and they could prove that low-loading fraction has a profound impact on the different properties of the nanocomposites [39,40,41,42,43,44].

According to the above literature survey, PMMA is the optimal candidate as a denture base material. However, its mechanical and tribological properties need further improvement. The main objective of the present study is to evaluate the effect of incorporating low fractions (0, 0.2, 0.4, 0.6, and 0.8 wt.%) of HA NPs on the mechanical properties (Young’s modulus, compressive yield strength, elongation, relative toughness, and hardness) and the tribological properties of PMMA. The tribological tests were conducted by rubbing the prepared nanocomposites against stainless steel and PMMA counterparts. A finite element model was constructed and used to evaluate the stresses generated from the friction in the tribological experiment. Finally, the morphological and structural properties of the PMMA nanocomposites and the rubbed surfaces from the friction tests were examined with X-ray diffractometry (XRD) and scanning electron microscopy (SEM) to understand the wear mechanism.

## 2. Experimental

As the PMMA resin, we used the Acrostone Special Tray Material (Cold Cure, Acrostone Dental & Medical Supplies, Cairo, Egypt), which comes in two separate compounds: a white PMMA powder, which is the primary polymer (density of 1.18 g/cm^3^), and a colorless MMA liquid, which is the monomer (density of 0.94 g/cm^3^). The HA NPs (NanoTech for Photoelectronics, Cairo, Egypt) were in the form of a white powder with a density of 3.02 g/cm^3^, a rod-like shape, and dimensions of 100 ± 5 nm (L) and 20 ± 3 nm (D).

To prepare the samples, the dry PMMA and HA powders were weighted to obtain specified weight ratios: zero, 0.2, 0.4, 0.6, and 0.8 wt.%. Then, they were mechanically stirred together for 5 min for the homogenous dispersion of the HA NPs in the PMMA powder, followed by the addition of the liquid monomer with different solid/liquid weight ratios (5–3.5). The weight of the resulting solid powder was calculated by summing those of the PMMA and HA powders. Next, the mixture was stirred manually at 28 °C and a relative humidity of 55%. When it became sticky like a dough (after around 20–30 s of mixing), the mixture was cased in a 25 mm × 8 mm cylindrical die through compression molding with a pressure of 14 MPa; after 30 min, the nanocomposites were hardened entirely and, thus, they were removed from the molds. These procedures were conducted according to the PMMA manufacturer recommendations and instructions [24].

**Figure 1:**
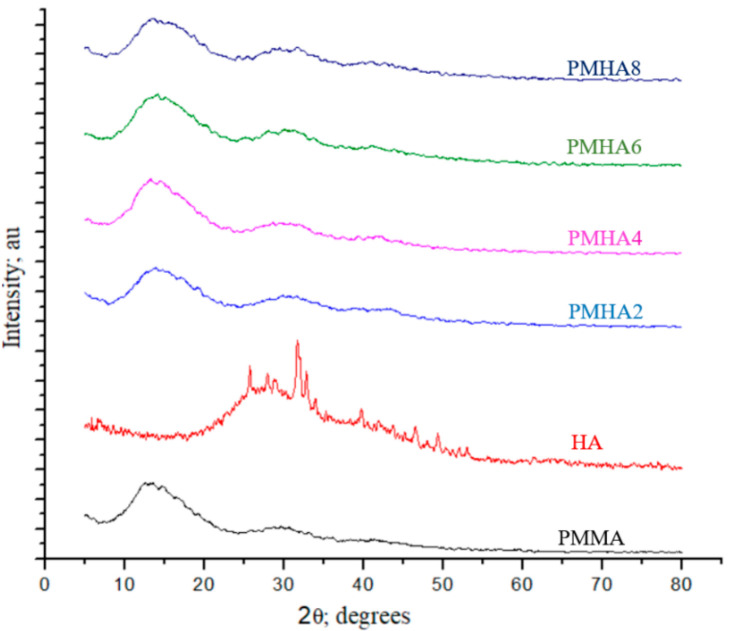
X-ray diffraction patterns of pure poly(methyl methacrylate) (PMMA), hydroxyapatite (HA), and PMMA/HA composites.

The PMMA/HA nanocomposites produced with HA weight fractions of 0, 0.2, 0.4, 0.6, and 0.8 wt.% were labelled as PMHA0, PMHA2, PMHA4, PMHA6, and PMHA8, respectively. Their chemical composition was determined via XRD analysis, as shown in Figure 1. The neat PMMA sample exhibited three main, broad XRD peaks, namely, a high-intensity band at 13.6° and two low-intensity ones at 30.7° and 41.8°; these peaks, which indicate the amorphous nature of the PMMA polymer [45], were also detected in all the PMMA/HA nanocomposites prepared. These results are identical to those reported by Ansari et al. [46] for PMMA. The XRD pattern of the HA NPs revealed a sharp peak, showing their crystalline phase, consistent with a previous study by Venkateswarlu et al. [47]. The XRD patterns of the PMMA/HA nanocomposites suggested an amorphous nature, indicating that the structural properties of PMMA were not affected by the HA incorporation and that no chemical reaction occurred between the PMMA and HA.

To evaluate the quality of the fabricated nanocomposites, their theoretical and experimental densities were compared; the theoretical density (*ρ_CT_*) was calculated based on the weight fractions and densities of the three components, according to the American Society for Testing and Materials (ASTM) standard test [48] as follows:

(1) $$\rho_{CT}=\;\frac{1}{\left(\frac{W_{P}}{\rho_{P}}+\frac{W_{H}}{\rho_{H}}+\frac{W_{m}}{\rho_{m}}\right)},$$

where *ρ_P_*, *ρ_H_*, and *ρ_m_* are the densities (in g/cm^3^) of PMMA, HA, and the MMA monomer, respectively, and *W_P_*, *W_H_*, and *W_m_* are their corresponding weight fractions.

The experimental density (*ρ_CE_*) was measured following the Archimedes approach [39]. The nanocomposites were weighed in air and alcohol, and then, their density was estimated as follows:

(2) $$\rho_{CE}=\left(\rho_{alc}-\rho_{air}\right)\times \frac{m_{Cair}}{m_{Cair}-m_{Calc}}+\rho_{air},$$

where *ρ_alc_* and *ρ_air_* are the densities (in g/cm^3^) of alcohol and air, respectively, and *m_Cair_* and *m_Calc_* are the corresponding nanocomposite masses (in g) in them.

The density measurement process was repeated six times for each nanocomposite sample, and the average values were taken. When comparing the theoretical and experimental values, the void volume fraction (*P_v_*) resulting from the fabrication methodology was estimated as follows:

(3) $$P_{v}\left(\%\right)=\;\frac{\rho_{CT}-\rho_{CE}}{\rho_{CT}}.$$

The mechanical properties of the PMMA/HA nanocomposites were assessed via hardness and compression tests. The hardness was estimated based on the shore hardness D index by using a durometer with a capacity of 5 ± 0.5 kg and a dwell time of 15 s, based on the ASTM D2240 [49]. It was measured six times along the nanocomposite surface, and the average hardness was determined, considering the standard error. Then, the samples were prepared and tested on a computer-controlled servo-hydraulic universal testing machine with a capacity of 30 tons and a strain rate of 1 mm/min; the stress-strain curves were measured, and the mechanical properties (Young’s modulus, relative toughness, relative ductility, and compressive yield strength) were estimated.

**Figure 2:**
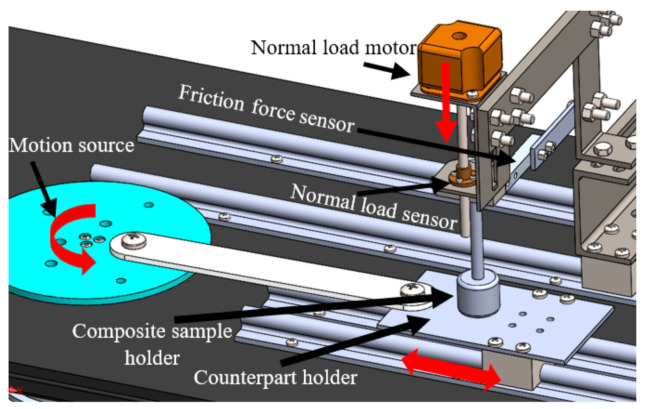
Reciprocating pin-on-disk tribometer.

The tribological characteristics of the PMMA/HA nanocomposites were investigated under dry sliding conditions at 27 °C and a relative humidity of 60% by utilizing a reciprocating pin-on-disk tribometer and a 50-mm stroke according to ASTM G99-95 [50], as shown in Figure 2. The PMMA/HA sample acted as the tribometer pin, with an 8-mm diameter and a 25-mm length, sliding against a rectangular disk made of stainless steel or PMMA. This test was conducted to simulate the real conditions of PMMA when used as a dental restorative material since friction can occur between the PMMA contained in different teeth in the mouth [51]. Furthermore, sometimes parents use stainless steel crowns to protect the teeth of their children from caries [52]; therefore, the friction and wear resulting from rubbing PMMA nanocomposites against stainless steel counterparts were also investigated. The surface roughness of these stainless steel and PMMA disks was 0.025 and 0.018 µm, respectively. Before each experiment, their surface was cleaned using acetone and then desiccated with a heat gun to eliminate any contaminant. In addition, the nanocomposite samples were washed ultrasonically and dried before the measurements.

The friction tests were conducted at a constant sliding speed of 0.4 m/s with different normal loads (3, 6, 9, and 12 N). The wear was calculated based on the difference in the sample weight before and after the experiment. For reliable results, each measurement was performed six times under the same conditions, and then the average values and standard errors were calculated.

After these tests, the morphology of the rubbed surfaces was inspected utilizing an SEM microscope (JCM-6000Plus; JEOL, Tokyo, Japan); for this analysis, all the surfaces were coated with a thin film of platinum to enhance their conductivity.

## 3. Results and Discussion

**Figure 3:**
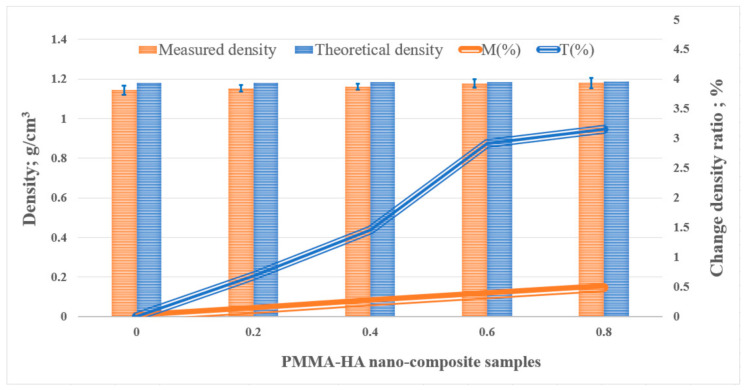
The density of the poly PMMA/HA nanocomposites.

Figure 3 compares the theoretical and experimental densities of the PMMA/HA nanocomposites, showing a visible difference since the measured values were lower than the calculated ones. This discrepancy could be attributed to the voids and pores formed during the nanocomposite fabrication: when PMMA, HA, and the liquid monomer were mixed manually outside the vacuum chamber, the chances of void formation increased. Furthermore, during the nanocomposite curing process, a high temperature is reached and, consequently, part of the monomer evaporates, further increasing the possibility of generating voids [10]. Incrementing the void fraction inside the nanocomposites can affect their various properties [53]. Consequently, the void volume fraction of the samples calculated using Equation (3) did not exceed 3% at 0.8 wt.%, which is acceptable [54]. This low presence of voids might be attributed to the compression molding step and the low HA loading, which preserved the coherence among the PMMA molecules.

The nanocomposite density slightly increased along with the HA weight fraction, in particular, by 0.5% and 3.25% for the theoretical and measured values, respectively. This result probably occurred because the low HA loading used did not exceed 0.8 wt.%. Such a negligible increase in the density of the final product preserves the wide applicability of the lightweight PMMA [55], including in dentures.

**Figure 4:**
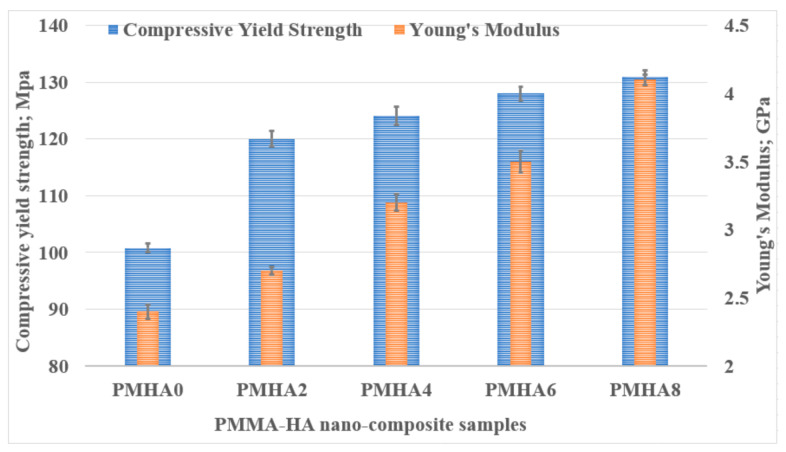
Young’s modulus and compressive yield strength of the PMMA/HA nanocomposites with different HA weight ratios.

PMMA is famous for its brittleness and usage as a denture base material, which exposes it to compression loads; hence, evaluating the compressive properties of PMMA/HA nanocomposites is essential. Figure 4 illustrates the average values of the measured Young’s modulus and compressive yield strength, showing the apparent increase in the Young’s modulus after raising the weight fraction of the HA NPs. The elasticity modulus of PMHA8 (4.1 GPa) increased by 70.8% compared with pure PMMA (2.4 GPa). Furthermore, the compressive yield strength gradually rose along with the HA nanoparticle loading, reaching an increment of 29.96% for PMHA8 compared with pure PMMA. Compared with previous studies on the effect of reinforcing PMMA with high loadings of HA NPs, which reported a deterioration in the compressive yield strength [36], these results prove that a low loading with such fillers can enhance the mechanical parameter instead.

**Figure 5:**
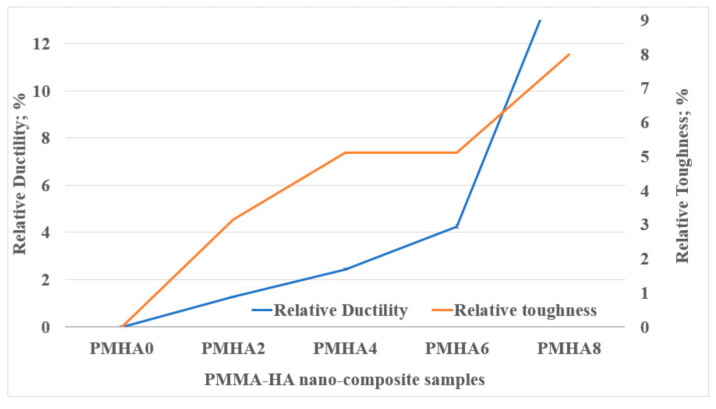
Relative toughness and ductility of the PMMA/HA nanocomposites with different HA weight ratios.

Figure 5 shows the relative ductility and fracture toughness of the PMMA/HA nanocomposites. Compared with pure PMMA, the ductility increased up to 9% when loading 0.8 wt.% HA nanoparticles. This demonstrates that the HA addition can transform the PMMA matrix from a brittle material into a ductile one. This outcome can be attributed to the energy absorbed by the HA nanoparticles under compression forces, limiting the crack propagation [56]. Moreover, the PMMA/HA nanocomposites exhibited a higher fracture toughness than pure PMMA, probably due to the brittleness of the latter. This indicates that the HA nanoparticles act as impact modifiers, in which it exhibited ductile fracture performance. In addition, an influence of the HA weight fraction on the ductility and toughness performance on the HA weight fraction was observed since they depend on the bonding between matrix and filler. The gradual increase in the ductility and toughness of the nanocomposites could be attributed to the low loading of the HA nanoparticles. Adding large amounts of fillers can decrease the fracture toughness because it reduces the homogeneity of the mixture and makes it weaker [57]. Chow et al. [37] reported that high weight fractions of HA (5, 10, 15, and 20 wt.%) could drastically decrease the fracture toughness. Therefore, a certain filler fraction could significantly affect the mechanical properties of the PMMA matrix. The comparison between these previous studies with the present results indicates that a low HA loading, instead, can enhance the mechanical properties of PMMA-based dentures.

**Figure 6:**
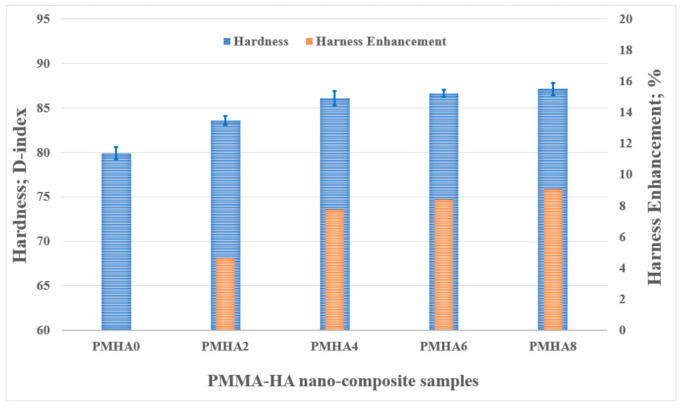
Hardness of the PMMA/HA nanocomposites with different HA weight ratios.

Figure 6 displays the variation in the nanocomposite hardness according to the HA loading, showing its gradual increase along with the weight fraction of the filler. PMHA8 exhibited the maximum hardness (87.7 D index), with an increase of 9% compared with pure PMMA (79.9 D index). The hardness of a composite depends on the strength of the intermolecular bonds between the nanoparticles and matrix; thus, its enhancement could be attributed to the uniform distribution of the HA nanoparticles inside the PMMA matrix [58], which promoted a good interface between them that enhanced the load transfer and consolidated the resistance against shear stresses resulting from volume compression [59]. The obtained results further demonstrate that a low HA loading can enhance the mechanical properties of PMMA, unlike high filler loadings that encourage agglomeration and deteriorate the material properties [60].

**Figure 7:**
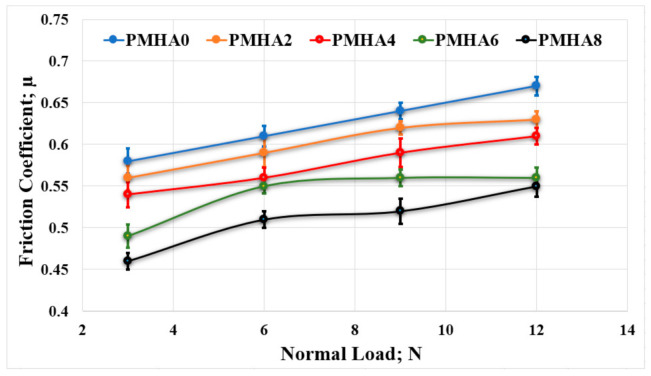
Friction coefficient of the PMMA/HA nanocomposites, with different HA weight ratios, rubbed against a stainless-steel surface under different normal loads.

Figure 7 shows the average friction coefficient, measured in the friction tests, as a function of the normal applied load, while rubbing the PMMA/HA nanocomposites against a stainless-steel counterpart. In all the cases, the addition of HA nanoparticles decreased the friction coefficient compared with pure PMMA. At a normal load of 3 N, PMHA8 exhibited the lowest friction coefficient (0.46), which is a reduction of 20.7% with respect to the pure PMMA (0.58). Under higher loads, the reduction in the friction coefficient between PMHA0 and PMHA8 ranged from 16% to 18%. Figure 7 also shows a gradual increase in the friction coefficient with the increase in the applied load; this can be due to the rise in temperature at the contact area between the rubbing surfaces [61], which can affect the adhesion between them [62].

**Figure 8:**
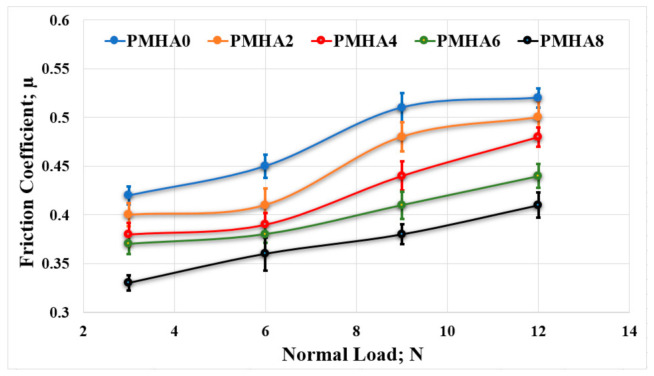
Friction coefficient of the PMMA/HA nanocomposites, with different HA weight ratios, rubbed against a PMMA surface under different normal loads.

Figure 8 illustrates the results obtained when rubbing the samples against a PMMA surface, showing a tribological performance similar to that against the stainless-steel counterpart. Under a normal load of 3 N, PMHA8 exhibited the lowest friction coefficient (0.33), with a 21.4% reduction compared with pure PMMA (0.42). However, the maximum difference (25.5%) between PMHA0 and PMHA8 was recorded when applying a load of 9 N. Moreover, the friction coefficient similarly increased along with the applied load.

**Figure 9:**
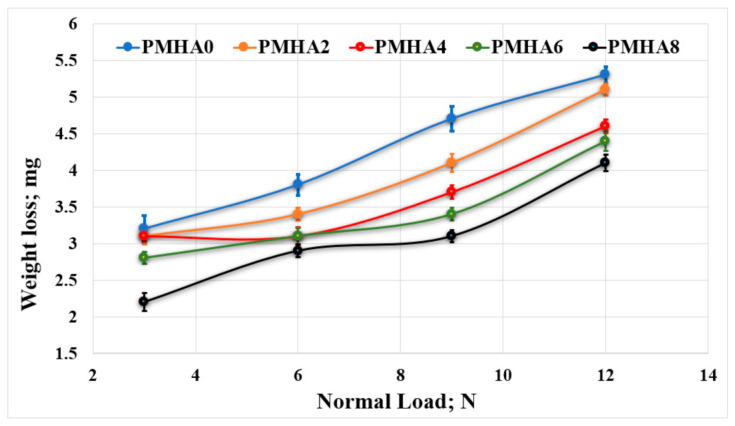
Wear of the PMMA/HA nanocomposites, with different HA weight ratios, rubbed against a stainless-steel surface under different normal loads.

**Figure 10:**
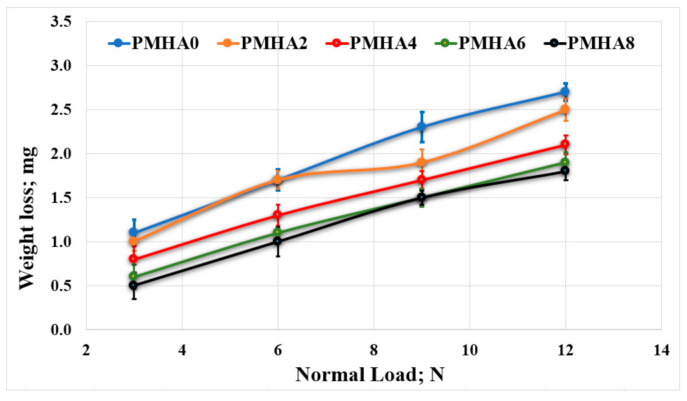
Wear of the PMMA/HA nanocomposites, with different HA weight ratios, rubbed against a PMMA surface under different normal loads.

Figure 9 and Figure 10 show the effect of the HA nanoparticle incorporation on the wear occurring during the tribological tests, revealing that increasing the HA weight fraction reduced the weight loss of the nanocomposites. These results indicate that the wear resistance increased when increasing the weight fraction of the HA nanoparticles. This could be attributed to the correspondingly enhanced mechanical properties, as discussed above; thus, the increased strength of the bonding between HA NPs and the PMMA matrix improved the load-carrying capacity, consequently limiting the degradation of the sample surface during the test [39].

Furthermore, the increase in the nanocomposite hardness by increasing the HA loading raised, in turn, the wear resistance of the fabricated material [38]. However, increasing the applied normal load could elevate the weight loss. This probably occurred because of the resulting higher temperature, which increased the frictional force, leading to the nanocomposite surface breakdown.

**Figure 11:**
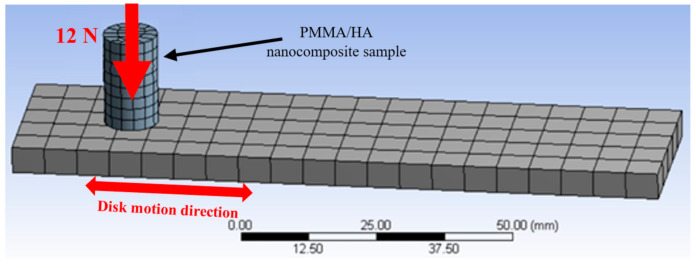
Finite element model of the friction test, including the poly(methyl methacrylate) (PMMA)/hydroxyapatite (HA) nanocomposite, rubbed against a stainless steel counterpart.

As mentioned above, the decrease in the friction coefficient and weight loss resulted from the enhancement in the load-carrying capacity of the nanocomposites after the HA addition. The load-carrying capacity can be evaluated by measuring the contact stress generated along the nanocomposite surface during the friction test [63]. Consequently, in the current study, a finite element model for the reciprocating frictional test was constructed by utilizing the explicit dynamics package of the ANSYS software, as shown in Figure 11.

The counterpart was modeled in the shape of a parallelogram (120 mm × 30 mm × 10 mm) and the PMMA/HA sample as a cylindrical pin (8-mm diameter and 15-mm height); the contact between them was defined as frictional to estimate the stresses resulting from the friction test. The counterpart meshes were created automatically by the software package, which meshed them into elements with hexahedron and tetrahedron shapes, for a total of 282 elements and 1810 nodes. The following boundary conditions were applied: the PMMA/HA nanocomposite was fixed in the x and y directions, and a normal force of 12 N was applied on its surface along the z-direction. The experimentally determined mechanical properties of the different samples were input in the software. The selected counterpart composition was stainless steel, with a reciprocating motion with a linear speed of 0.4 m/s and a stroke of 50 mm.

**Figure 12:**
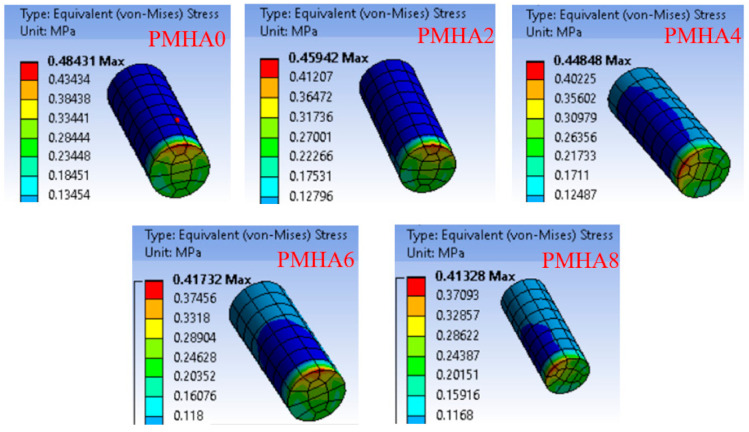
Equivalent stress distribution along the surface of the PMMA/HA nanocomposites, with different HA weight ratios.

**Figure 13:**
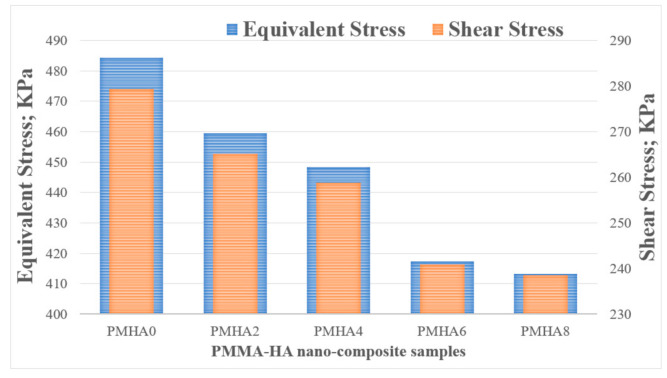
Equivalent and shear stresses on the surface of the PMMA/HA nanocomposites, with different HA weight ratios.

Figure 12 shows the distribution of the contact equivalent stress on the surface of the various PMMA/HA samples. The stress was concentrated at the surface edge in all the cases, probably due to the movement direction. The HA incorporation reduced the maximum equivalent stress on the nanocomposite surface. These results could be attributed to the demonstrated enhancement in the nanocomposite strength of the nanocomposites, and consequently increased load-carrying capacity and decreased friction coefficient [64]. Figure 13 displays the generated shear stress as a function of the HA nanoparticle loading, showing a reduction of approximately 15% when increasing the filler content, corresponding to a decreased friction coefficient [33].

**Figure 14:**
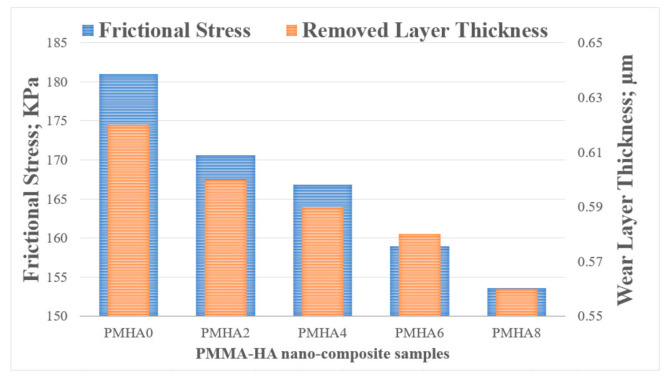
Frictional stress and wear layer thickness on the surface of the PMMA/HA nanocomposites, with different HA weight ratios.

As shown in Figure 9, the weight loss during the friction test decreased with increasing the HA weight fraction. Thus, in the finite element analysis, the wear layer thickness and the generated friction stress were estimated as shown in Figure 14, indicating an agreement between simulated and experimental results. Increasing the HA weight fraction decreased the shear stress on the sample surfaces and the frictional stress between the rubbing surfaces; hence, the wear layer thickness decreases, resulting in a reduced weight loss of the nanocomposites.

**Figure 15:**
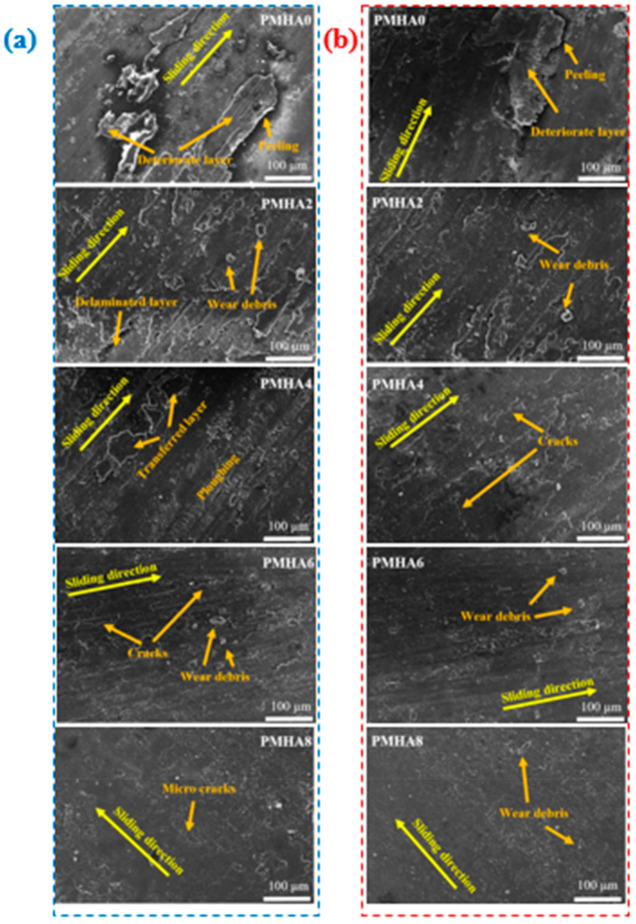
Scanning electron micrographs of the surface of the PMMA/HA nanocomposites, with different HA weight ratios, after being rubbed against (**a**) stainless steel or (**b**) a PMMA counterpart.

Both the experiments and simulations showed the dependence of the PMMA/HA wear on the HA weight fraction. Therefore, the wear mechanism induced during the friction test was examined via SEM observation, as shown in Figure 15, revealing that the morphology of the rubbed surfaces varied accordingly with the HA loading. PMHA0 exhibited many deteriorated layers and peeling due to the ploughing of its rubbed surface, which in turn led to an increase in the weight loss. Furthermore, the eliminated weak layers can increase the shear resistance and, consequently, the friction coefficient [65]. Moreover, the brittle failure of the rubbed surfaces incremented their roughness, decreasing the toughness. The delamination wear mechanism, which usually raises the friction coefficient and wear rate [66], was dominant in the case of pure PMMA. The surface morphology of the other PMMA/HA nanocomposites, instead, appeared relatively smooth; this could be attributed to the enhancement in the nanocomposite strength and hardness by increasing the HA loading. As a result, there were fewer deteriorated layers, reducing the weight loss and friction coefficient.

PMHA2 and PMHA4 exhibited a fatigue wear mechanism, revealed by the appearance of some microcracks and wear debris due to the induced ploughing. Increasing the HA NP loading to 6 and 8 wt.% facilitated the stress transfer between the PMMA matrix and HA NPs; therefore, the weight loss decreased, and the ploughing effect almost disappeared. As a result, PMHA6 and PMHA8 showed considerably fewer microcracks and wear debris, as well as a surface smoothness that reduced the shear force and friction coefficient.

## 4. Conclusions

The effect of low HA nanoparticle loading in PMMA for dentures to enhance its mechanical and tribological properties was investigated. The results demonstrated that increasing the HA weight fraction up to 0.8 wt.% can increase the hardness, Young’s modulus, compressive yield strength, ductility, and fracture toughness by 9%, 70.8%, 29.9%, 13%, and 9%, respectively, compared with pure PMMA, while ensuring only a negligible change in the density. The tribological analysis showed a reduction of 20% and 25% in the friction coefficient against stainless steel and PMMA counterparts, respectively, when adding 0.8 wt.% HA nanoparticles. The wear resistance also increased along with the HA weight fraction under various normal loads. A finite element model proved that the load-carrying capacity was enhanced by the HA incorporation. Finally, the morphology of the rubbed surface indicated that adding HA nanoparticles into a PMMA matrix can change the wear mechanism and decrease the weight loss during a friction process. In the future, the authors will try to investigate the effect of the low-loading fraction of micro HA particles and compare it with the addition of HA nanoparticles.

## Data Availability

The data presented in this study are available on request from the corresponding author.
